# Experiences in a Clinical Innovation Pharmacy Fellowship: A Novel Model of Ambulatory Care Training and Practice Advancement

**DOI:** 10.3390/pharmacy13010028

**Published:** 2025-02-17

**Authors:** Alison Doane, Nicholas Cox, Shannon Gadd, Erin Gurney, Payson Ashmead, Kyle Turner

**Affiliations:** 1 Medical Group, University of Utah Health, 127 500 E, Salt Lake City, UT 84102, USA; alison.doane@utah.edu (A.D.); payson.ashmead@pharm.utah.edu (P.A.); 2 College of Pharmacy, University of Utah, 282 2000 E, Salt Lake City, UT 84112, USA; nicholas.cox@pharm.utah.edu; 3 School of Medicine, University of Utah, 30 N 1900 E, Salt Lake City, UT 84132, USA; shannon.gadd@hsc.utah.edu; 4 Intermountain Healthcare, 5121 Cottonwood St, Murray, UT 84107, USA; erin.gurney@imail.org

**Keywords:** ambulatory care, fellowship, leadership, practice development

## Abstract

The University of Utah Clinical Innovation Fellowship models novel partnerships between third-party payers, clinical practices, and academia. While healthcare costs continue to increase unabated and physician burnout leads to provider shortages, this fellowship focuses on both crises by training pharmacists to establish new practices in ambulatory clinic spaces using funding provided by third-party payers. Not only does this fellowship represent a future in which pharmacists are able to address third-party payers’ need to reduce healthcare costs and clinics’ need to address provider shortages, it also successfully trained fellows to pursue jobs in ambulatory care and academia. Payers, clinics, providers and patients all expressed a high degree of satisfaction with the work of the fellows. In multiple clinics where fellows established new pharmacy services, those services led directly to new job approvals funded by the clinics themselves. The purpose of this paper is to serve as a model by which fellowship programs elsewhere can be designed, as well as to show that partnerships between ambulatory clinics, payers, and pharmacists are both sustainable and beneficial to all parties including, most importantly, the patients who receive better care for their complex chronic disease states. While this paper is descriptive in nature, work is ongoing to objectively measure the impact of the fellows on patients, providers, and third-party payers. A sampling of outcomes is presented, describing the impact of the pharmacist fellows’ efforts to improve medication management in primary care. Even with limited objective measures of success, we are able conclude that over the past 3 years, the fellowship has accomplished its aim of preparing fellows for future roles in ambulatory care, practice design, and academia while also demonstrating that a funding model aligning payers, clinics, and academia is sustainable.

## 1. Introduction

In response to the growing crises of physician shortages and escalating healthcare costs [1,2,3], and in an effort to advance practice within the state, faculty at the University of Utah College of Pharmacy created the Clinical Innovation Fellowship. It is already known that pharmacists integrated in the primary care setting can reduce provider burnout by sharing the workload, improving providers’ ability to prescribe medications for chronic diseases, increasing patients’ capacity to meet health goals, and improving the overall management of patients [4]. In this way, it was hypothesized that the creation of the Clinical Innovation Fellowship at the University of Utah would meet the needs of and provide proactive solutions for healthcare systems and primary care providers at a critical junction between payers, health systems, and a college of pharmacy. Additionally, it was hypothesized that the creation of the fellowship could also create a novel path of continued training for postgraduate year 1 (PGY1) trained pharmacist trainees. Organizations like the American College of Clinical Pharmacy (ACCP) recognize the need for more specially trained pharmacists that provide high-quality patient care, along with the challenges of expanding pharmacy residency programs to train more of these pharmacists [5,6]. As more pharmacists pursue specialty training [7], there is a need to create novel training methods, such as this fellowship, to meet this demand.

This fellowship is designed to create solutions to these problems by establishing sustainable pharmacy services in an ambulatory care setting. A pharmacist in this fellowship role serves as a connection between patients, their providers, and the payer. By serving all three parties, a pharmacist is uniquely positioned to impact the quality and cost of healthcare, all while supporting providers who are overburdened and burnt out. Further, pharmacists are provided with the skills needed to advance pharmacy practice throughout their careers by completing the fellowship. This paper describes the development of the Clinical Innovation Fellowship by the University of Utah and provides a template for others to develop their own programs.

## 2. Materials and Methods

### 2.1. Program Description

The Clinical Innovation Fellowship has three primary objectives: (1) establish ambulatory care pharmacy services at a large multi-specialty clinic and through this gain experience in ambulatory care pharmacy and practice development/innovation, (2) determine/describe the value of pharmacy services in the ambulatory care setting by collecting data before and during the implementation of comprehensive medication management (CMM), and (3) gain competence in the four pillars of academia including scholarship, ambulatory care practice, professional service, and teaching. The fellowship is designed as a one-year training program for an individual who has completed a PGY1 residency and has experience in the ambulatory care setting. Founded in 2020, this fellowship includes one fellow position per year and involves partnerships with the University of Utah College of Pharmacy, third-party payers, and private practice sectors to provide innovative ambulatory care pharmacist services in novel settings. While similar residencies or fellowships have been developed [8], this is the first that we are aware of that involves a relationship with third-party payers. By involving payers, this fellowship differentiates itself from other programs via its ability to create sustainable pharmacist positions that generate revenue for clinics using payer funds. The fellow, in turn, gains valuable skills in practice by developing pharmacy services in new arenas to advance the profession. While “fellowship” is a broad term used to describe various pharmacy practice and research programs, a fellowship provides the flexibility needed for the novel collaboration between a payer, private clinics, and a college of pharmacy. The fellowship directors designed a series of suggested objectives for the program, with the flexibility to adjust these based on the fellow’s areas of interest.

### 2.2. Fellowship Design

While the fellowship was designed to be flexible to meet the individual fellow’s professional goals and interests, the fellow must meet the objectives of the fellowship to graduate. These objectives are organized into five domains: Practice, Scholarship, Teaching, Service, and Professional Development. Specific activities the fellow could expect to participate in were then assigned to each domain, creating a well-rounded experience that prepares the fellow for a variety of career paths in pharmacy practice and academia. Table 1 gives an overview of the expected activities required to graduate from the fellowship and a more detailed list of activities completed by each resident can be found in Appendix A (Table A1).

### 2.3. Practice Experience

To accomplish the above, fellows were placed at a primary care practice that had not had pharmacists before. The primary care sites chosen were staffed by a multidisciplinary team including a physician, advanced practice clinicians such as the physician’s assistants and nurse practitioners, as well as medical residents and medical students. These clinicians were trained in various fields including internal medicine, pediatrics, family medicine, and internal medicine/pediatrics. The fellow’s role throughout the fellowship would be to establish pharmacist services in that environment. Before establishing a new practice, the fellow worked at a University of Utah primary care clinic, which already had established pharmacist services. This clinic served as a model upon which the fellow could base their own practice. After a sufficient onboarding period at the clinic, flexible to the needs of the fellow, they would begin spending 3 days per week establishing their new clinical practice. To continue to develop clinical skills and gain experience in a successful practice, the fellow would continue to serve as the pharmacist at the University clinic once per week. This clinical time also allowed the fellow to experience a layered learning model, as this clinic frequently hosted both medical and pharmacy learners.

### 2.4. Other Learning Domains

**Figure 1:**
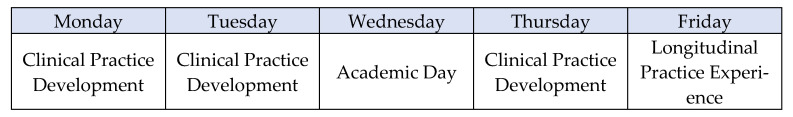
Weekly schedule of the day-to-day activities of the fellow.

One day per week, the fellow completed various projects aligned with their interest areas that fulfilled the fellowship’s objectives. This day is referred to as an “academic day” and was utilized in whatever way best served the productivity of the fellow. Unlike residencies, additional weekend/evening staffing was not included in the fellowship. Figure 1 shows an example of the weekly design of activities.

### 2.5. Fellowship Oversight

The fellowship directors, both of whom have a combined 18 years of practice development and academic experience, served as coaches and mentors to the fellows as they established their new practices. They also created tailored learning experiences in the other fellowship domains based on the interests of the fellow. A shared administrative model for the two directors was created in order to leverage the strengths, interests and relationships of each director. Directors engaged the fellow in mentoring meetings every other week and practice site visits at least twice throughout the year. Additional meetings could be accommodated at the request of the fellow or fellowship directors as needed. Because the two fellowship directors shared a primary care practice site at the University of Utah, the oversight of this longitudinal practice was split between the directors. Once a quarter, a more formal evaluation was conducted between both directors and the fellow to discuss in, greater detail, the achievements, progress, and opportunities for growth/improvement in the fellowship.

### 2.6. Program Funding

The funding for the fellowship position, housed within the College of Pharmacy, was a 50–50 model between the school and the payer. This way, the College of Pharmacy would bear the responsibility of hiring, paying and performing various human resource functions for the fellow each year while the payer, in addition to providing 50% of the funding, could focus on identifying and providing the fellow with lists of patients most in need of pharmacy services. In this agreement, the payer recognized the benefit of implementing a clinical pharmacist in their primary care practice and received this benefit at a discounted rate as they provided only part of the position’s funding and the fellow’s salary was less than that of a clinical pharmacist post-training. Ultimately, the payers were investing in a strategy that was improving patient care for at-need populations. However, all stakeholders recognized that an improvement in financial and clinical outcomes likely could not be observed in the scope of a 1-year fellowship due to the nature of the training program. The selection of the payer partner was based on long-standing relationships and the interest of each payer’s leaders. Fellowship directors also negotiated a practice site that had a high percentage of patients covered by the third-party payer, with a particular focus on private practices not affiliated with major health systems. Ultimately, practice sites interested and engaged in value-based models became the ideal sites for fellows to develop pharmacy services. Additionally, the fellow provided a unique contribution to teaching, precepting, and scholarship for the College of Pharmacy. No funding was provided by the clinic where the fellow was placed to establish pharmacist services; however, the clinic played a critical role by serving as a pilot site.

### 2.7. Fellowship Assessment

The outcomes of the fellowship were based on the interest of the individual fellow, their ability to gather data, and the abilities and interests of the practice site and payer. The goal of the fellowship was to create added value for all parties and evolved from year to year through a process of collaboration and continuous program improvement. For this reason, samples of a variety of the outcomes describing the impact that the participating fellows had on their clinical practice locations and the career success they have enjoyed post-fellowship will be presented in this paper. Some outcomes that will be discussed include the medication therapy problems (MTPs) identified by the Pharmacy Quality Alliance (PQA), provider satisfaction, patient satisfaction, practice site sustainability, and fellow job placement upon graduation.

The PQA criteria for MTPs were used in order to promote the consistent categorization and coding of MTPs and the related actions to resolve the MTPs [9]. The framework builds upon categorizing each actionable MTP into one of four categories of MTPs (in order of preference; I = Indication, E = Effectiveness, S = Safety, and A = Adherence). Then, an intervention (Outcome) is associated with each MTP. If multiple MTPs were identified for a single drug, only the highest level/preference MTP was coded.

Patient and provider satisfaction were measured after one year of the fellowship via a validated survey and qualitative assessment of provider interviews. The specific methodology and results of this study have been published previously [10]. Provider satisfaction was determined via interviews, similar to the method used by Funk et al. [11]. Meanwhile, patient satisfaction was assessed via a validated patient satisfaction survey developed and validated by Moon et al. [12].

Practice site sustainability was measured based on whether the pharmacist position started by the fellow was supported, formally created, and funded beyond the duration of the fellowship. This was tracked in order to show value to the payer and practice site, and to measure whether the pharmacy practice could grow and be sustained in private practice.

The job placement of the graduating fellow was measured in terms of whether the graduating fellow found employment upon graduation. This was tracked in order to show that this unique training model could result in trainees that are qualified, in a competitive market, for jobs in ambulatory care pharmacy.

## 3. Results

At the time of publication, three fellows have successfully graduated from the fellowship program over the three years it has been offered. All three fellows had already completed a PGY1 residency, although each entered the fellowship with different residency experiences.

In one academic year of the fellowship, a fellow identified 1106 individual MTPs. In order of priority, Indication made up 79 (7%) of the MTPs, with 21 (2%) attributable to “unnecessary drug therapy” and 58 (5%) attributable to “needs additional medication therapy”. Effectiveness made up at least 9% of the MTPS, with 98 (9%) attributable to “effectiveness”. Similarly, Safety made up at least 10% of the MTPS, with 112 (10%) attributable to “adverse medication event”. The majority of the MTPs identified fall into the final category of Adherence, with 443 (40%) related to “adherence” and 176 (16%) related to “cost”. Regrettably, 198 (18%) MTPs were unable to be coded due to there not being enough detail in the tracking mechanism. The tracking of MTPs also reflects the growth of the fellow as a pharmacist over the course of the fellowship, with an individual fellow documenting 20 MTPs during the first month at the practice site and 163 MTPs in the final month.

Previously published findings showed that the fellow, as an embedded clinical pharmacist providing comprehensive medication management (CMM) at a private primary care clinic, had a positive impact on both provider and patient satisfactions [9].

The fellowship was able to meaningfully advance the practice of pharmacy locally after the first year of the program, resulting in a full-time pharmacist position being fully funded by the clinic. Two of the three fellowship positions resulted in full-time pharmacist positions being created to fill the role being left by the fellow upon graduation. During their fellowship, the individuals delivered a high level of patient care through the use of collaborative practice agreements, allowing them to manage chronic disease states requiring considerable amounts of provider follow-up, such as diabetes and hypertension. Based on the fact that positions were created for these pharmacists upon fellowship completion, they provided significant value to the practice site that was worthy of funding the role of a full-time pharmacist.

All of the fellows were employed as full-time ambulatory care pharmacists, practicing in primary care, immediately after graduating from the fellowship. One of the fellows was hired at a private clinic, while the other two fellows were hired at academic medical centers, directly competing with postgraduate year 2 (PGY2) trained pharmacists for their positions. Upon graduation, in addition to competing in a highly competitive local ambulatory care pharmacist job market, the fellows were uniquely qualified for these positions through the development experiences they likely would not have experienced through traditional pharmacy residencies.

## 4. Discussion

The strengths of the experience include the novel funding mechanism that requires buy-in from payers, which is a more financially feasible model of funding pharmacist positions than positions funded entirely by a clinic, given the low to non-existent revenue that a pharmacist historically generates. In addition to providing a unique experience via the establishment of new pharmacist services, the fellowship also provided training for fellows that enabled them to compete with other pharmacists trained in ambulatory care for jobs within a clinic. The most similar type of learning environment to this fellowship is a PGY2 ambulatory care pharmacy residency. When compared to a PGY2 ambulatory care residency program, this fellowship has some distinct advantages and disadvantages. The advantages include a greater ability to customize experiences to the individual fellow’s interest, a greater focus on research and academia than a residency allows, and the enhanced independence of the fellow in gaining experience and initiating pharmacy services in a novel setting. The disadvantages include a lack of a formal accreditation, which may be viewed negatively by potential employers, a reduced focus on precepted clinical training, and the challenge of integrating a non-traditional learner into a health-system that is organized around resident learners (e.g., preceptor schedules, co-resident socializing, etc).

The fellowship also provided significant exposure to academia. At the time of submission, one fellow is employed by a Physician Assistant program at a School of Medicine teaching pharmacotherapy and leading the development of new student-run clinics. Upon hire, the hiring faculty specifically cited the experience the fellow gained within the College of Pharmacy, as well as their experience teaching, publishing, grant-writing, and serving on various committees, as making them a qualified and desirable candidate for the position. Another fellow is an adjunct faculty member at a College of Pharmacy, which they attribute directly to their academia experience within the fellowship program.

Finally, the payers and value-based companies involved in the funding of the fellowship also benefited from the better management of their most costly patients, further showing that our novel funding model is a mechanism by which pharmacists moving forward may fill a significant gap in our healthcare delivery system.

There are some weaknesses in this evaluation of the fellowship program. Most notably, we do not have specific data to report regarding patient/provider satisfaction (other than that which is already published [10]), its impact on healthcare costs from the payer perspective, or an objective measure of the reduced burden on primary care providers by adding clinical pharmacy support. While this is a significant limitation in the evaluation of this fellowship, there are some key findings that signal satisfaction from the parties involved. The Clinical Innovation Fellowship still maintains a high level of investment among the funders (payers and the College of Pharmacy), and the work of the fellows led to the creation of new pharmacist positions funded by the clinics they were placed in, signaling support from clinic leadership as well. While these subjective measures indicate that this fellowship was a successful endeavor, future evaluations of the fellowship will focus on data collection over a longer period of time, using objective measures, to justify its existence. Specifically, in addition to patient and provider satisfaction and a quantitative analysis of the fellows’ work, future iterations could focus on reducing the number of provider visits and other objective drivers of primary care provider (PCP) burnout.

## 5. Conclusions

The Clinical Innovation Fellowship was developed to explore the novel relationship between pharmacists in primary care, colleges of pharmacy, and insurance payers to create sustainable solutions that justify the cost of their services and improve access to safe and effective medication use. The fellows effectively identified and managed medication therapy problems, had a positive impact on both patient and provider satisfaction, created jobs for ambulatory care pharmacists, and trained additional pharmacists that were qualified for the same roles as traditionally PGY2 trained pharmacists.

## Figures and Tables

**Table 1:** Overview of activities required for fellowship graduation.

Domain	Requirement
Practice	Clinical skills enhancement in established practice experience (1 day/week)
	Clinical practice development at new site (3 days/week)
Scholarship	Submit 1 grant proposal
	Conduct 1 primary research project, concluding with poster and manuscript submission
	Give 1 state-level CE presentation
	1 CoP presentation on research project(s) to faculty/staff
	Submit 2 presentation for consideration at national conferences
	Submit 3 different manuscripts, which could include: Primary fellowship project Project in collaboration with other faculty Smaller-scale project in collaboration with other pharmacy learners (e.g., help mentor PGY1 or pharmacy student research project)
Teaching	Serve as a coursemaster for a class or specific class module
	Serve as facilitator for weekly small-group classroom sessions for pharmacy students for 1 semester
	Give 3 lectures to pharmacy students
	Precept and plan rotation experiences for pharmacy students at established practice site
Service	Formal membership on a CoP committee (e.g., Curriculum Committee)
	Other optional tasks State organization committee involvement National organization committee involvement Volunteering as a preceptor at a student-run free clinic Student organization precepting
Professional development	Participate in 2 meaningful development programs that correspond to one of the 4 pillars of academia. Examples include: Professional teaching seminar Enrolled in graduate-level course on biostatistics Enrolled in graduate-level course on teaching in higher education Institutional professional development courses Institutional leadership courses CoP grant-writing workshops Attend 1 national conference
	Leadership discussions with fellowship directors

Abbreviations: CE = Continuing Education, CoP = College of Pharmacy.

## Data Availability

Research data is not currently available to share.

## Appendix A

**Table A1:** Elements of the fellowship completed by each individual fellow.

Domains and Experiences	Fellow 1	Fellow 2	Fellow 3
Practice	University of Utah Primary Care Granger Medical Clinic	University of Utah Primary Care Granger Medical Clinic	University of Utah Primary Care Intermountain Health Primary Care
Scholarship	1 Grant submission State level CE presentation CoP presentation on research project National Presentation National Presentation and Preceptor’s Conference abstracts submission 1 Article published 1 Manuscript published	1 Grant submission 2 State level CE presentation CoP presentation on research project National presentation submission Primary project (Granger) Tenure/Research Faculty project Smaller-scale project(s)	State level CE presentation CoP presentation on research project Poster Presentation at a national meeting 1 Manuscript: Pending
Teaching	Integrated therapeutics lecture at college of pharmacy Recitation facilitator Delivered 5-6 CoP Pharmacy lectures Precepted 9 APPE students and 2 PGY1 residents	Recitation Facilitator Foundations of Patient Centered Care CoP lecture Advanced therapeutics debrief Community Practice CoP lecture Skills Lab precepting Precepted Students and PGY1 residents	Co-coursemaster elective at Utah College of Pharmacy Foundations of Patient Centered Care CoP lecture Skills lab precepting Integrated therapeutics lecture CoP lecture Recitation facilitator at CoP Precepted Students and PGY1 residents
Service	CoP Assessment committee Local organization Board National Committee Research Committee	CoP Admissions Committee Precepting at local student run clinic	CoP Curriculum Committee Precepting at local student run clinic
Professional Development	Attended 2 national meetings Attended 1 national webinar series Completed 1 national Teaching seminar	Completed 1 national teaching seminar University of Utah Trainer Essentials Course Completed a Biostats course	University of Utah Trainer Essentials Course Attend 1 national meeting Completed a Teaching in Higher Education Course

Abbreviations: CoP = College of Pharmacy.
